# Widely tunable and high resolution mid-infrared laser based on BaGa_4_Se_7_ optical parametric oscillator

**DOI:** 10.1007/s12200-023-00077-0

**Published:** 2023-09-26

**Authors:** Qing Ye, Hui Kong, Jintian Bian, Jiyong Yao, Enlong Wang, Yunlong Wu, Haiping Xu, Kaihua Wen, Yihua Hu

**Affiliations:** 1 State Key Laboratory of Pulsed Power Laser Technology, National University of Defense Technology, Hefei, 230037 China; 2 Advanced Laser Technology Laboratory of Anhui Province, Hefei, 230037 China; 3 Technical Institute of Physics and Chemistry, Chinese Academy of Sciences, Beijing, 100190 China

**Keywords:** Widely tunable, High resolution, BaGa_4_Se_7_, Optical parametric oscillator

## Abstract

The widely tunable and high resolution mid-infrared laser based on a BaGa_4_Se_7_ (BGSe) optical parametric oscillator (OPO) was demonstrated. A wavelength tuning range of 2.76–4.64 μm and a wavelength tuning resolution of about 0.3 nm were obtained by a BGSe (56.3°, 0°) OPO, which was pumped by a 1064 nm laser. It is the narrowest reported wavelength tuning resolution for BGSe OPO, and was obtained by simultaneously controlling the angle and temperature of BGSe.

**Graphical Abstract:**

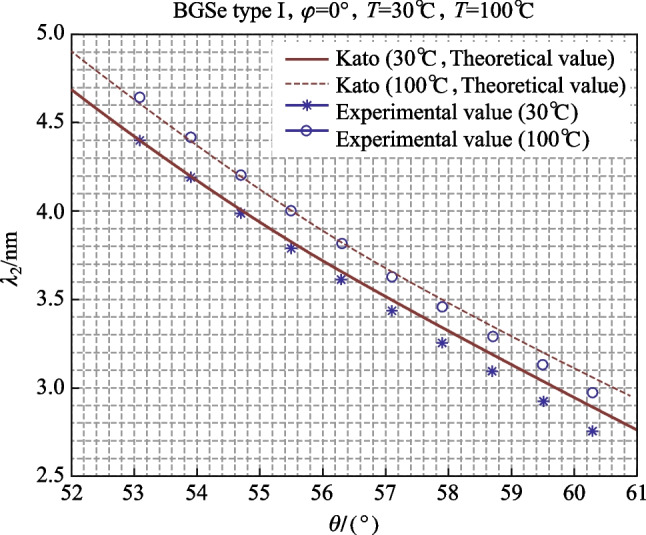

## Introduction

Widely tunable and high resolution mid-infrared radiation sources operating in 3–5 µm region have been applied to numerous frontier applications, including remote sensing, molecular spectroscopy, and atmosphere environmental monitoring [1, 2]. Taking atmospheric monitoring as an example, due to the narrow absorption peak linewidth of atmospheric molecules, it is necessary to strictly align the output wavelength with the target wavelength, which requires high wavelength resolution. Optical parametric oscillation (OPO) is an attractive approach to achieving this, especially when high energy and average power are demanded simultaneously [3, 4]. The newly developed mid-infrared crystal BaGa_4_Se_7_ (BGSe) exhibits a wide bandgap (2.64 eV), wide transparent range (0.47–18 µm), and high laser damage threshold (557 MW/cm^2^), which is beneficial for OPOs pumped by an economical 1064 nm laser [5, 6].

In 2010, the BGSe crystal was synthesized for the first time [5]. In 2013, Yang et al. demonstrated a mid-IR optical parametric amplifier with BGSe pumped by a 1064 nm Nd:Y_3_Al_5_O_12_ (Nd:YAG) laser, and a 3–5 μm idler tuning range was demonstrated for the first time. The angle of BGSe was tuned from 51.5° to 58°, corresponding to the average $\Delta \lambda /\Delta \theta$ of 309 nm/° [7]. In 2015, a 6.4–11 μm idler tuning range was demonstrated. The angle of BGSe was tuned from 41° to 46°, corresponding to the average $\Delta \lambda /\Delta \theta$ of 920 nm/° [8]. In 2016, Kostyukova et al. achieved unprecedented tuning capability from 2.7 to 17 μm with a single crystal cut by angle tuning [9]. This is the broadest range of tuned laser output for BGSe OPO. The average $\Delta \lambda /\Delta \theta$ was 680 nm/° (The angle of BGSe was tuned from 40° to 61°). In 2020, Yang et al. achieved an 8–14 μm range wavelength tuning, and the average $\Delta \lambda /\Delta \theta$ of 1395 nm/° (The angle of BGSe was tuned from 40.2° to 44.5°) [10]. In 2022, Tian et al. achieved 3.7–17 μm range wavelength tuning by using femtosecond laser pumping [11]; the average $\Delta \lambda /\Delta \theta$ was 831.25 nm/° (The angle of BGSe was tuned from 58° to 42°). In those articles, researchers used angle tuning to achieve the wavelength tuning of BGSe OPO. In 2021, Kong et al. used temperature tuning to achieve wavelength tuning of BGSe OPO for the first time [12]. In 2023, Yang et al. achieved a high conversion efficiency of 9.3–10.6 μm range wavelength tuning with BGSe crystal temperature varying from 45 °C to 5 °C, corresponding to an average wavelength adjustment of 30.85 nm/°C [13].

In the above experiments, for angle tuning, a larger wavelength tuning range could be obtained, but there were two shortcomings.

1. The tuning resolution was not high. Taking the article [9] as an example, the $\Delta \lambda /\Delta \theta$ was relatively small in mid-infrared, compared to the far-infrared band. When the angle of BGSe crystal was tuned from 60° to 50° under type I phase matching conditions, the output wavelength was from 3 to 5 μm. The average wavelength change rate with angle was 200 nm/°. When the angle of BGSe crystal was tuned from 50° to 40° under type I phase matching conditions, the output wavelength was from 5 to 17 μm. The average wavelength change rate with angle was 1200 nm/°. The output wavelength resolution depended on the angle resolution of the angle tuning device in the experiment. The angle resolution was not provided in Ref. [9], we assumed that the resolution of the angle tuning device was 0.01°, so the wavelength change rate was 2 nm/0.01° in mid-infrared, and 12 nm/0.01° in far-infrared. The wavelength change rate was smaller under type II-B phase matching conditions, but it reached 1.5 nm/0.01° (4–8 μm @ 20°–46°).
2. The output wavelength shifted with changes in ambient temperature. According to the article [12], at an output peak wavelength of 3.5 μm, the output wavelength rose 3.2 nm when the temperature of BGSe rose 1 °C.

To solve the above problems, we propose to tune the temperature and angle of BGSe crystals simultaneously, placing BGSe crystals in a temperature-controlled furnace, and then placing the temperature-controlled furnace on an electric rotary platform. This scheme can achieve both wide-range wavelength tuning and high resolution wavelength tuning with stable output wavelength. It also overcomes the problem that the output wavelength of BGSe OPO shifts with changes in ambient temperature.

A peak wavelength of 3.6 μm with an angle tuning range of 2.76–4.64 μm, was obtained by a 1.06 μm pumped laser. The wavelength tuning resolution was 0.3 nm, which is the narrowest reported resolution, and the output wavelength is stable.

## Experimental setup

**Fig. 1:**
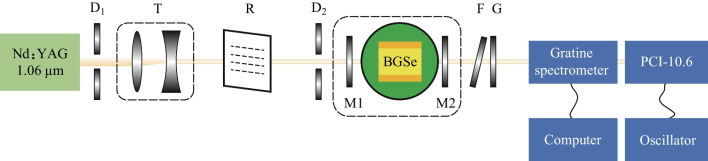
Schematic diagram of the experimental setup

The experimental setup is shown in Fig. 1. The BGSe OPO was pumped by an SL800 Series pulsed Nd:YAG laser, with 13 ns pulse width (FWHM), 8 mm beam diameter, and 1 Hz pulse repetition frequency. A pinhole was placed behind the Nd:YAG for adjusting the light path, and the beam diameter was compressed to 4 mm through a telescope system to improve the energy density of the pump light.

A 90° optical rotator, R (in Fig. 1) could change the polarization direction of the pump light from horizontal polarization to vertical polarization.

**Fig. 2:**
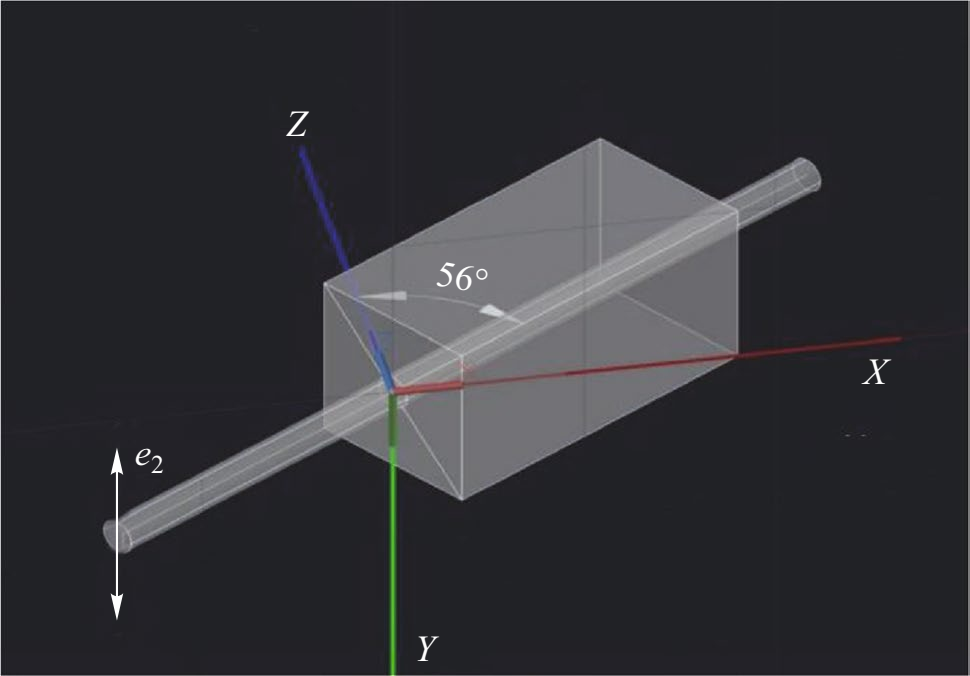
Dielectric (*XYZ*) frames of BGSe

As shown in Fig. 2, in order to tune the wavelength over a wide range, it was necessary to change the angle* θ*, which was between the crystal *Z* axis and the direction of the incident light. To meet the phase matching condition, the pump light should be *e*_2_ light; that is, the polarization direction of the pump light was perpendicular to the *XOZ* plane of the crystal. So the *XOZ* plane was horizontal. In that case, tilting the crystal along the horizontal direction could change the* θ* of BGSe, and the crystal would not slide from the temperature-controlled furnace. If the 90° optical rotator was not used in this experimental setup, then *XOZ* would lie in a vertical plane, so that changing the *θ* of BGSe would cause sliding of the crystal from the temperature-controlled furnace easily.

An electric rotator (Zolix TBR100, angle resolutioin: 0.01°) was placed between the M1 and M2 mirrors, and a temperature-controlled furnace (HCP TC038-PC, maximum temperature: 200 °C, temperature resolution: 0.1 °C) was placed on the electric rotator and fixed with a connector made by 3D printing. BGSe was held in the temperature-controlled furnace by a copper gripper.

Due to the large size of the electric rotary table and temperature-controlled furnace (100 and 70 mm), the length of the OPO cavity was long. So the pump threshold of OPO was relatively high. To prevent the pump light from damaging the crystal, we used a 3D printer to make a self-made OPO cavity mirror bracket, which reduced the cavity length to 93 mm.

M1 and M2 were highly transmissive (HT) for the pump (P, *t* > 95%) and highly reflective (HR) for the signal (S, 1.35–2.06 μm, *R* ≈ 85%), and HT for the idler (I, 2.3–5 μm, *t* > 90%). The BGSe crystal was polished and HT for the pump, signal, and idler lights.

A filter and a Ge plate were placed behind M2. The transmittance of the filter was about 1% at 1064 nm and 95–99% at 3–5 μm. The transmittance of Ge was zero at 1064 nm and about 80% at 3–5 μm. The idler light was detected by a grating spectrometer (Omni300*λ*, Zolix). The peak wavelength of the blazed grating is 3000 nm, the grating groove density was 300 g/mm, and the minimum resolution was 1 nm. The computer controlled the rotation of the grating to make its transmission wavelength tunable from 2000 to 4800 nm, and the adjustment accuracy was 1 nm. The Vigo PCI-10.6 was used to detect the idler light transmitted from the grating spectrometer. The idler light energy from PCI-10.6 was measured by a DSOX3054 oscilloscope. When the maximum energy emerged in the oscilloscope, the wavelength set by the grating spectrometer was the peak wavelength of the idler light.

## Results and discussion

### Wide range of angle tuning at 30 °C

Four experts have given Selleimer equations for BGSe at room temperature: BadiKov [14], Yang [8], Boursier [15], and Kato [16] in 2011, 2015, 2016, and 2017, respectively. According to these four equations, four different phase matching curves can be obtained. Several articles have compared the experimental idler wavelength of BGSe OPO at room temperature with the four theoretical values of idler wavelength, but most of them have not given the specific temperature of the crystal during the experiment. According to Ref. [12], the output wavelength of BGSe OPO changes with ambient temperature. To prevent the output wavelength of BGSe OPO from changing with temperature, we used a temperature-controlled furnace to stabilize the temperature of BGSe crystal at 30 °C, then adjusted the output wavelength by changing the angle of the crystal.

According to the equation in Ref. [17] that BGSe refractive index changed with temperature, the Sellmeier equation given by BadiKov [14], Yang [8], Boursier [15], and Kato [16] when room temperature was modified to 30°C, and their phase matching curves at 30 °C were given by the modified Sellmeier equation. The above calculation method can be referred to Ref. [12].

**Fig. 3:**
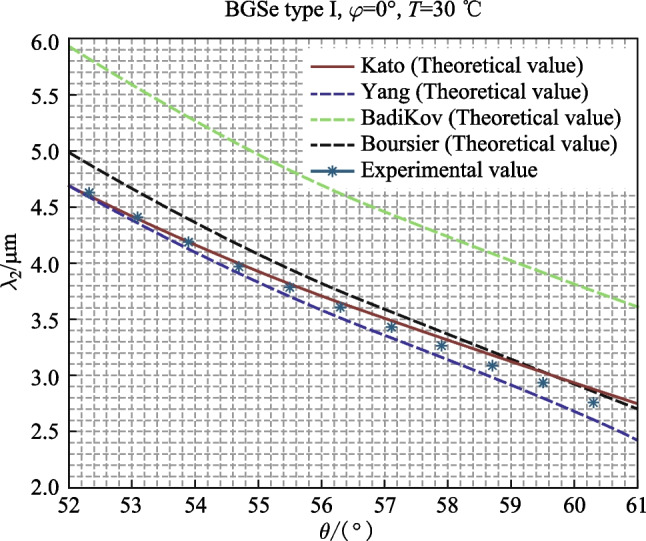
Relationship between BGSe OPO idler wavelength and angle

According to the law of refraction, when the crystal rotates 10° relative to the incident light, the angular variation of the refractive light inside the crystal is about 3.98° (*n*_BGSe_ ≈ 2.5). The idler wavelength was measured with output pulse energy of about 0.4 mJ, which corresponded to a pumping beam energy of about 15.2 mJ. The experimental and theoretical values of the output wavelength of the BGSe crystal at 30 °C with an external rotation angle of − 10° to + 10° (the internal variation was 52.32° to 60.28°) are shown in Fig. 3.

As shown in Fig. 3, the experimental values were relatively close to the Sellmeier equations of Kato and Yang. At 4–4.6 μm, the theoretical value of Kato is close to being consistent with the experimental value.

### Small range angle tuning at 30 °C (56.2°–56.3° in steps of 0.01°)

**Fig. 4:**
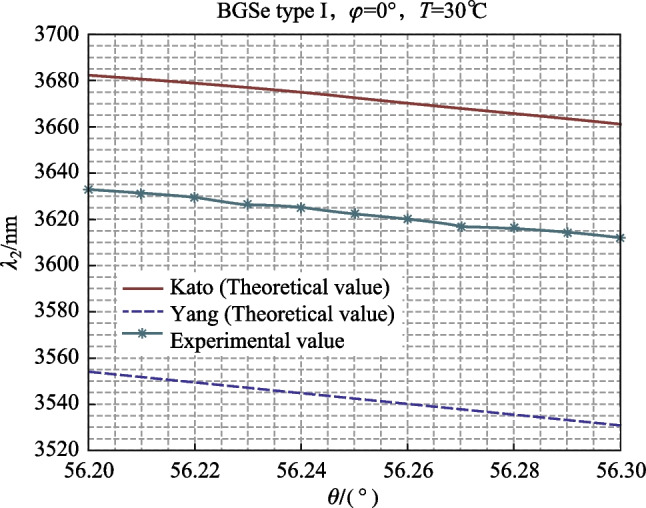
Relationship between BGSe OPO idler wavelength and fine angle adjustment

At 30 °C, we changed the angle of the electric rotator to change the output wavelength. The relationship between the experimental values and the theoretical values of Kato and Yang is shown in Fig. 4.

As shown in Fig. 4, when the crystal angle was tuned from 56.20° to 56.30° at 30 °C, the output wavelength decreased from 3633 to 3612 nm, and the rate of change of wavelength with angle was 2.1 nm/0.01°. The theoretical rates of change of wavelength with angle calculated by Kato and Yang were 2.1 and 2.3 nm/0.01°, which is close to agreement with the experimental value.

According to the above experimental results, when the temperature is fixed, a stable wavelength output can be obtained, with a wavelength tunning resolution of approximately 2.1 nm at the peak wavelength of 3.6 μm (assuming an angle resolution of 0.01°). To obtain a narrower resolution laser wavelength output, we simultaneously performed angle and temperature tuning on BGSe OPO.

### Temperature tuning at different angles (at 30 °C and 100 °C)

**Fig. 5:**
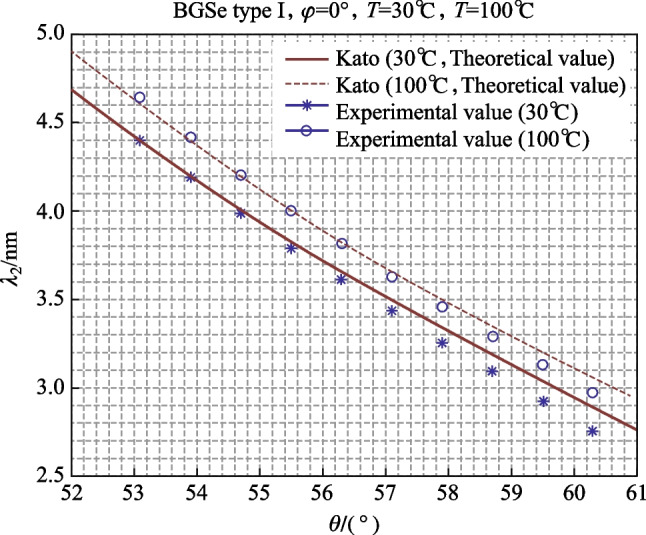
Relationship between output wavelength and angle and temperature

We rotated the crystal angle to between 53.1° and 60.3° (10 increments with intervals of 0.8°), and then raised the crystal temperature from 30 °C to 100 °C. The heating effects can cause a damage to the crystal in case of high-power optical pumping. For the safety of the BGSe crystal, we raised the temperature from 30 °C to only 100 °C, instead of the maximum of the oven, which is about 200 °C. The theoretical and experimental values are shown in Fig. 5.

As shown in Fig. 5, when the temperature rose from 30 °C to 100 °C, the wavelength of idler light variation ranged from 60.3°, 59.5°, 58.7°, 57.9°, 57.1°, 56.3°, 55.5°, 54.7°, 53.9°, and 53.1° were 2765–2978, 2932–3136, 3096 − 3297, 3263–3463, 3435–3635, 3612–3819, 3797–4006, 3990–4206, 4192–4419, and 4410–4646 nm, respectively. The maximum output wavelength at each angle point was larger than the minimum output wavelength at the next angle point, so continuous wavelength tuning from 2765 to 4646 nm could be achieved. In addition, at 10 angles point, the output wavelength increased by 236, 227, 216, 209, 207, 200, 200, 201, 204, and 213 nm, respectively, with a wavelength change rate of 2.85–3.37 nm/°C. Therefore, the output wavelength tuning resolution should be 0.285–0.337 nm (assuming a temperature solution of 0.1 °C).

### Temperature tuning at fixed angle (*θ* = 56.3°)

**Fig. 6:**
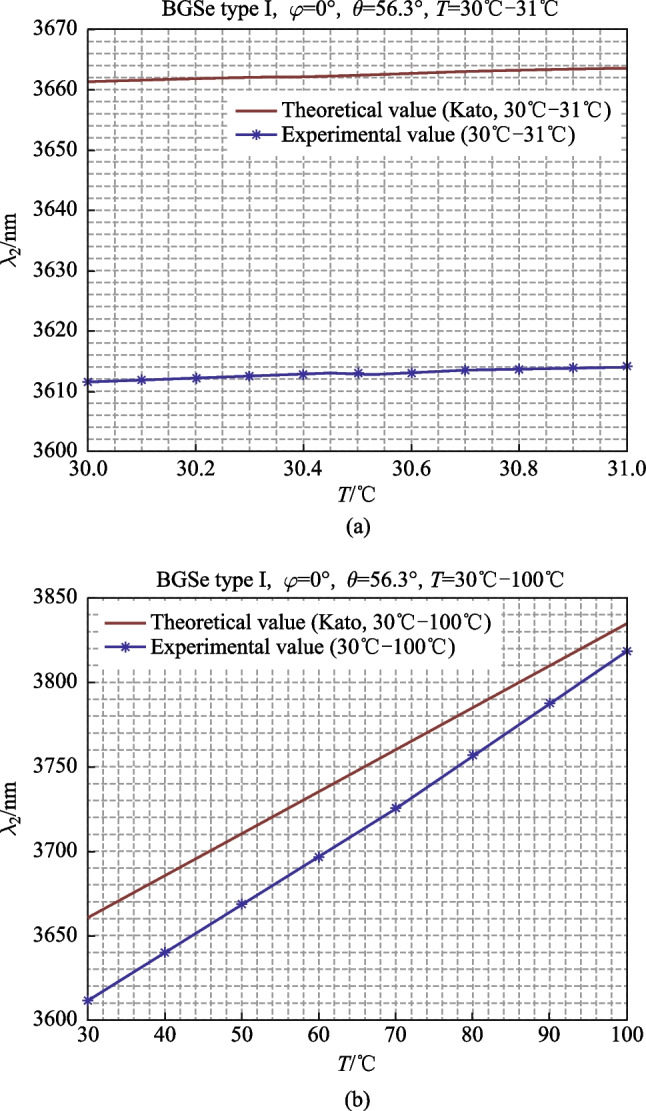
Relationship between output wavelength and temperature rose **a** from 30 °C to 31 °C, and **b** from 30 °C to 100 °C

At normal incidence (*θ* = 56.3°), the crystal temperature is increased from 30 °C to 31 °C with a temperature changing step of 0.1 °C. The relationship between the output wavelength and temperature was measured as shown in Fig. 6a. Due to the small range of temperature change at this time, the change of output wavelength was less than 1 nm. To obtain a more accurate output wavelength value, it is necessary to use data fitting to determine the output wavelength. The Gauss function obtained by fitting was

$$y=a\times \mathrm{exp}\,{(-\frac{x-b}{c})}^{2},$$

where the peak wavelength was *b*, and the output linewidth (FWHM) was $2\sqrt{\mathrm{ln}2}\times c$.

The method for accurately fitting the output wavelength was given in Ref. [18].

At normal incidence (*θ* = 56.3°), the temperature of the crystal is increased from 30 °C to 100 °C, with a temperature changing step of 10 °C. The relationship between the output wavelength and temperature is shown in Fig. 6b.

As shown in Fig. 6a, when the temperature rose from 30 °C to 31 °C at *θ* = 56.3°, the idler wavelength of theoretical output was 3661.2 to 3663.6 nm, and the rate of change of the idler wavelength with temperature is 0.24 nm/0.1°C. The experimental value shows that the idler wavelength increased from 3611.5 to 3613.9 nm, with an idler wavelength rate of change of 0.24 nm/0.1 °C. The theoretical and experimental values agreed well, indicating that the developed BGSe OPO had a stable and high-resolution wavelength tuning ability.

As shown in Fig. 6b, when the temperature rose from 30 °C to 100 °C at *θ* = 56.3°, the theoretical output wavelength was 3661.2 to 3834.5 nm, and the rate of change of the output wavelength with temperature is 2.47 nm/°C. The experimental value shows that the idler wavelength increased from 3612 to 3819 nm, and the rate of change of the output wavelength with temperature was 2.96 nm/°C. The slope of the measured wavelength was higher than that of the theoretical value, and the output wavelength did not linearly increase with the temperature, but had a small curvature.

When the temperature was higher, the rate of change of the output wavelength was larger. For example, the wavelength increased by 28 nm when the temperature changed from 30 °C to 40 °C, while the wavelength increased by 31 nm when the temperature changed from 90 °C to 100 °C.

In our previous work, we have measured the output spectral width of BGSe OPO and narrowed its linewidth. When the center wavelength of the output laser was at 3.5 μm, its output spectral width was about 4.5 nm, which was slightly higher than that of KTA OPO, but much smaller than that of ZGP OPO. When we used the method of inserting an FP etalon into the L-type OPO cavity to narrow the spectral width of the output of BGSe OPO, the narrowest linewidth of 1.27 nm could be obtained [18].

When FP etalons with other parameters are used, we could expect to obtain a narrower linewidth in mid-infrared and far-infrared laser output (below 0.5 nm). Then, combined with the wide-band tuned and high-resolution mid-and far-infrared laser output method proposed in this paper, it can have a more significant application prospect.

## Conclusion

We controlled the temperature and angle of BGSe OPO simultaneously, and a widely tunable range, high resolution mid-infrared laser was obtained. The wavelength tuning range was 2.76–4.64 μm, and the wavelength tuning resolution was around 0.3 nm, which is the narrowest resolution. Compared to single angle tuning, adding a temperature-controlled furnace could ensure that the output wavelength did not change with ambient temperature, therefore maintaining a stable output wavelength. In addition, the addition of temperature control also improved the output wavelength tuning resolution. The wavelength tuning resolution was changed from 2.1 (resolution of the rotator was 0.01°) to 0.3 nm (resolution of the temperature controlled furnace was 0.1 °C).

The tuning range and step size of the angle (from 60.3° to 53.1° with 0.8° steps) and temperature (from 30 °C to 100 °C with 0.01 °C step) were explored in the experiment, under which continuous tuning of wavelengths could be achieved. This scheme has considerable reference significance for the commercial development of BGSe crystals.

## Data Availability

The data that support the findings of this study are available from the corresponding author, upon reasonable request.
